# The Therapeutics of Colic1Read before the Thirty-second Annual Meeting of the United States Veterinary Medical Association, Des Moines, Iowa, September, 1895.

**Published:** 1895-10

**Authors:** W. L. Williams

**Affiliations:** Veterinarian to the Agricultural Experimental Station, Bozeman, Montana


THE JOURNAL
OF
COMPARATIVE MEDICINE AND
VETERINARY ARCHIVES.

VOL. XVI. *OCTOBER*, 1895. No. 10.

### THE THERAPEUTICS OF COLIC.[1]

BY W. L. WILLIAMS,
VETERINARIAN TO THE AGRICULTURAL EXPERIMENTAL STATION, BOZEMAN, MONTANA.

THE term colic has been used to include a variety of diseases of the abdominal organs due to widely dissimilar causes and possessing but one common character, that of abdominal pain having its origin in some portion of the digestive canal. This grouping of a number of diseases under one term is partly necessary because many of them cannot be clearly differentiated during life, although all have characteristics in a measure peculiar to themselves which may lead to a probable or even positive diagnosis.

Statistics indicate that 10 to 20 per cent. of horses affected with colic die, and that 40 per cent. of the deaths of horses are due to this affection. These statistics appear to be largely from city districts, hence apply to work-horses. In breeding districts these figures are probably entirely too high.

The difficulty of surely identifying the various diseases of this group tends unconsciously to lead to the empirical treatment of the entire group by a common formula, regardless of specific indications offered by each individual case. Under these conditions we find the treatment of colic unsatisfactory. The Munich veterinary school (Friedberger and Fröhner, *Lehrbuch d. Spec. Path. u. Therap.*, vol. i., p. 162) has shown that an apparently greater per cent. of colic cases recover without than with medication. Have we not permitted empiricism to displace

[1] Read before the Thirty-second Annual Meeting of the United States Veterinary Medical Association, Des Moines, Iowa, September, 1895.

rational medicine to an unjustifiable degree? The conclusions of the Munich veterinary school are applicable to several veterinary colleges in our country, and to the practice of many veterinarians.

During my first years in practice, when I followed trustingly the methods dictated by authors and teachers, my losses from colic were appalling, costing me many anxious hours and valued patrons, but as necessity and reason drove me farther and farther from what I thought classic methods and taught me a higher appreciation of Nature's powers and her abhorance of unnecessary meddling in disease my losses became very much less. If we are to handle colic without violence to natural methods we must apply to it greater knowledge and judgment than to any other internal malady. The confusion in its treatment is shown by the endless number of remedial agents used without any apparent order. One will have the patient lie; another stand; another walk, or trot, or run. One relies on anæsthetics, another on sedatives, another on stimulants or carminatives, another upon astringents, and others upon laxatives or purgatives. Not a few combine several or many of them in inextricable confusion, or give together two drugs diametrically opposed, as opium with oil or aloes, in such proportions as partly or wholly to counteract each other.

For therapeutic reasons, among the diseases grouped under the common head of colic, because not always distinguishable from each other, we must recognize numerous pathological conditions due to various causes, among which are:

1. Dietetic colic.
    *a.* Overfeeding colic.
    *b.* Colic due to damaged or improperly-prepared food.
    *c.* Starvation colic.
2. Obstruction colic.
    *a.* Impaction colic, including the impaction of dry, woody food in large intestines of adults; the retained meconium or ingested straw or hay of young foals; and the gummy feces occasionally seen in horses at pasture or on green grass.
    *b.* Mechanical obstruction.
        1. Volvulus.
        2. Intussusception.
        3. Herniæ.
        4. Tumors.

3. Colic due to paralysis of the intestines.
4. Colic due to the interruption of the gastro-intestinal blood-supply.
    *a.* Thrombosis.
    *b.* Embolism.
5. Reflex nervous colic due to exposure, fatigue, and other causes—spasmodic colic.
6. Colic due to external mechanical pressure or to abnormal position of the body.
7. Worm colic.
8. Colic due to foreign bodies in the intestinal canal.
    *a.* Calculi.
    *b.* Sand or grit.

The application of rational therapeutics to this group of pathological conditions renders it necessary that our first law shall be rigidly to avoid the use of any medicinal agency which would tend to aggravate any pathological condition present, whether recognizable or not, or to pursue as far as possible an expectant line of treatment.

In the colic of overfeeding with its history we have no cause to suspect any obstruction, but have to deal with an overloaded gastro-intestinal canal, the contents being moist, soft, and tending to rapid decomposition with the formation of large quantities of gases, with the consequent dangers of gastro-intestinal ruptures, suffocation, intestinal paralysis, gaseous poisoning, and other untoward results.

The chief and most urgent element of danger is the gaseous accumulations, from which relief should be given as promptly as practicable for many reasons:

1. To avoid rupture of the stomach, intestines, or diaphragm.
2. To prevent suffocation.
3. To prevent toxic effects from the absorption of gases.
4. To permit normal peristalsis and avoid gastro-intestinal paralysis.
5. To relieve pain.
6. To prevent intestinal displacement.

The use of the trocar for relieving gaseous distention is so prompt, efficacious, and safe that no apology is necessary for mentioning this method almost alone. In order to accomplish fully all the purposes indicated above the use of the trocar is demanded as early as any appreciable degree of tympany is present, and its repetition as often as like conditions return.

The gaseous accumulations are generally most pronounced in the large colon and cæcum, and we usually aim to enter these with the trocar; the point usually selected for the operation is the highest part of the right flank, in which position, if the animal be standing, the gas is most free from admixture with food and consequently flows most freely through the canula. If the patient is lying down the intestines are quite probably displaced somewhat, but most likely the most distended intestine occupies the highest point of the abdominal cavity, whether that be the middle or lower region of the flank, right or left side.

There has been an endless variety of trocars invented, the simplest being distinctly the best. The calibre of the trocar usually kept for sale is too great, being $\frac{1}{8}$ inch diameter or more, while $\frac{3}{32}$ inch is ample and adds to safety by decreasing the area of the wound about one-half. This reduction in size necessitates the use of the best of steel, well tempered, so it will not bend. The point of the trocar should be far more tapering, the sharp part being two to three times as long as in those generally offered, and delicately sharpened, so that its use occasions an incised and not a punctured wound. The canula should fit the trocar closely and perfectly, leaving no space between them, and the end of the canula should be well rounded and smooth, so that no foreign matter can be lodged between the end of it and the trocar and carried into the tissues, nor the peritoneum or the intestine caught and pushed along before the canula. The trocar should be fastened immovably in the handle. Reversible trocars cannot be kept in order, their points always being dulled by contact with the handle when reversed. If the instrument is to be carried in the pocket it should be supplied with an appropriate case. A very serious defect in the construction of most reversible instruments is the making of the trocar too short for the canula, so that when used the set-screw fails to hold it in place, allowing it to push back into the handle so far that the point is partly hidden within the canula, allowing the end of the latter to tear and abrade the tissues, marring or wholly preventing a successful operation. The minimum net length of the canula should be six inches, and even this will be found short for very fat heavy draft-horses. If too short, the gut slips off the end very readily, either from peristaltic movement or collapse of engaged portion. Besides, it may be necessary to pass through one intestine and enter another part.

Before using, the trocar should be carefully sterilized, and when practicable the part to be selected for the operation should also be cleansed, although this is not generally so important, for if the trocar be properly made as described it cannot well drag foreign matter far into the tissues. The incision of the skin at the point of operation by means of a lancet is unsurgical, producing a larger and more open wound than that caused by the trocar, through which infection may the more readily occur. The skin serves as an important bulwark against septic infection and should be left as nearly intact as practicable, which is best done by using the small trocar with its sharp, well-tapered point, which cuts like a lancet and leaves, when withdrawn, a wound almost undiscoverable.

The trocar should be held loosely in one hand, perpendicularly to the abdominal walls, and its insertion accomplished by a quick stroke with the palm of the other hand, driving it to its desired depth at a single blow. The trocar is then withdrawn, and the canula is retained in position with the hand, being careful to prevent its dislodgement from the intestine by peristaltic action. After the gas has escaped the trocar should be reinserted into the canula, so that on withdrawal the intestinal walls catch and wipe off any ingesta which might otherwise adhere to the mouth of the canula and drop into the peritoneal cavity or lodge in the abdominal walls. More abscesses and septic inflammations are probably due to drawing septic material from the intestine and depositing it in the tissues than are caused by the introduction of septic material from without.

Should the escape of gas be unsatisfactory the depth of introduction may be varied by placing the trocar within the canula and pressing it forward or drawing it outward. Frequently it is found that a small amount of gas escapes from the intestine first puncture, when it collapses and the pressure of other portions of the intestine is such that the portion encountered does not refill. In such areas the collapsed part of the bowel is to be passed through and distended portion entered. If success is wanting on the right side there should be no hesitation in trying the left.

Fatal sequelæ from the use of the trocar have been recorded, but I have not observed them. In one case I produced peritonitis of a severe type, owing to too short canula from which the intestine repeatedly slipped and probably caused the lodgment of intestinal contents in the peritoneal cavity. In another case a very serious abscess took place in the abdominal walls, finally rupturing in the inguinal region. In this case the instrument was used in the dark, and the horse was thickly smeared with wet manure at the point of operation, the case being one of unusual urgency. Several times small abscesses have followed the use of the trocar, but they were insignificant and did not interfere with the daily work of the patient.

It has been suggested that when the stomach is the seat of considerable gaseous distention, as evidenced by attempts at vomition and eructations of gas, it should be attempted to introduce the trocar into it, and it has been claimed that this operation has been successfully performed by the use of an extra long trocar introduced at the usual point for entering the colon or cæcum, the practicability of the operation being explained upon the theory that when distended with gas the stomach turns on its longer axis and floats upward. But in autopsies which I have conducted after rupture of the stomach that organ was found in its normal position, and the position of the contents in the peritoneal cavity indicated clearly that the relation of the stomach had been constant. It is rare, however, that the stomach requires direct relief from gaseous distention, as the gases are most frequently formed in the intestines and pass forward into the stomach, or, being formed in the stomach, pass readily backward through the intestine until the colon is reached. The stomach does not rupture because the gas within it is under greater pressure than in other parts of the alimentary canal, but because of the much greater surface upon which the gaseous pressure is exerted at the same rate per unit of surface. If the colon be relieved by the use of the trocar the stomach is immediately relieved also in nearly all cases. At least, I have not observed rupture of this organ in any case where the colon was kept free from gaseous accumulations. Should emptying of the colon fail to relieve the gastric distention the stomach-tube may be found useful.

When the tympany has been relieved by the aid of the trocar the chief element of urgency has been overcome, and the pain and discomfort of the animal have been markedly alleviated, so the attendant has time to proceed deliberately and carefully. The patient should be made as comfortable as possible, and a good, deep bed of straw provided, upon which, if it will lie down, it should be allowed to rest quietly. If very restless and not exhausted, slowly walking sometimes allays the uneasiness and tends to hasten the evacuation of the bowels. The patient should be prevented from throwing itself violently on the ground.

Hot fomentations, poultices, brisk rubbing, or the application of stimulating liniments to the abdominal walls may be of value if the pain present demand it. The remedies to be administered should be so selected that, while alleviating the pain, they should not interfere with the normal secretion or peristaltic motion of the alimentary canal and that they should, as far as possible, check further decomposition of food and the consequent gaseous accumulations. Opium and morphine are both clearly contra-indicated, as they serve to constipate and paralyze the bowels and augment the gaseous distention. Turpentine, camphor, etc., relieve the pain fairly well, but are too prone to irritate.

Carminatives, such as ginger, anise, peppermint, etc., exercise a wholesome influence by stimulating the normal action of the bowels, especially when combined with alcohol or its derivatives, such as nitrous ether, these latter possessing in addition to their antispasmodic properties a distinctly antiferment action.

Chloral hydrate claims here a very important place, being markedly antiseptic yet anodyne in a high degree. Chloral has the disadvantage that after a full dose, especially in solution, it is dangerous to follow soon after with a drench, as the chloral so benumbs and anæsthetizes the throat and larynx that thereafter swallowing is imperfect and the medicine drops into the larynx and passes into the lungs, producing foreign-body pneumonia, more frequently in my observation acute lung-apoplexy, ending fatally in one to a few hours. Some writers, among them Finlay Dunn, state that chloral may be safely administered hypodermically, but after three exceedingly unfortunate attempts to use the drug in this way, in which extensive gangrene of the subcutaneous connective-tissue ensued, in each case disabling the patient for several weeks, I became convinced that the drug was not usable in this manner. Again, I have tried administering the drug in the dry form, wrapped in paper, but the paper may break in the mouth or fauces and cause painful excoriations, or may lodge in the œsophagus and cause inflammation of that organ, producing symptoms of choking. In one case to which I was called fifty miles in consultation—a supposed case of choking—I found on arrival that the œsophagus had been burned by the lodgment of the chloral pill. It is therefore best to give chloral in small, soft, tenacious pills which do not readily break, cannot lodge in the œsophagus, and once in the stomach will quickly melt away.

An important question confronting the veterinarian is the action to be taken regarding the presence in the intestinal canal of a superabundance of food. Generally purgatives are resorted to as the rational treatment indicated, but a careful study of the conditions presented and observation with the various methods of treatment demonstrate this to be erroneous. The ordinary purgatives act too slowly in the horse, so that ere purgation ensues the crisis has been safely passed, and if let alone the bowels will move gently or undergo slight diarrhœa.

In these cases of overfeeding with flatulence the bowels have been distended, more or less paralyzed, and are usually irritated or inflamed. A full dose of aloes under these conditions means that it cannot act until the colic ceases, or rather until the time when naturally the colic should cease, when in the weakened state of the bowels the colicky pains are renewed by the drastic action of the aloes, and when the bowels finally respond they do so violently, not infrequently ending in the death of the animal, quite commonly delaying the return of the horse to his accustomed duties for several days. In other cases aloes remains in the system for a long time causing great depression and in some cases fatal aloe-poisoning without showing any tendency to purgation.

The bland oils, while doing little if any good in these cases, do comparatively little harm, but the already overloaded stomach can certainly be benefited but little by their introduction. I have noted, also, that when pills wrapped in paper are given after oil has been taken and still remains in the stomach, the wrappers become oiled and the pill is not dissolved for several hours. In this way I have seen chloral pills repeated several times without effect, until finally the oil has been absorbed and then the combined effects of several large doses of chloral were manifested all at once.

Other purgatives, such as Glauber salts, tartar emetic, and calomel have been commended and are probably safer than aloes or oil, although their value may well be doubted.

More recently, pilocarpine and eserine have come into prominence as remedial agents in this affection, and like most new remedies have been much abused and have then tended to relapse into disuse. If eserine has a place in veterinary medicine it is certainly in this disease. The food is generally soft, and needs only prompt peristaltic action to cause its expulsion from the alimentary canal, and this eserine can furnish. Friedberger and Fröhner fear that in the overfilled condition of the stomach and bowels that the severe muscular contractions are likely to produce rupture. This would doubtless be true in cases of mechanical obstruction, such as volvulus, or of inflammation with consequent paralysis of a section of intestine, or of impaction with hard dry food, or in the presence of great accumulations of gases. The mechanical obstructions we rarely suspect in cases where a clear history of overfeeding exists; the impaction with hard dry feces rarely, if ever, occurs in these cases; the inflammation and paralysis of a section of the bowel usually take place only after several hours' duration of the disease, and the gaseous distention is subject to our control by surgical means. We therefore see no direct danger in the use of eserine in the earlier stages of this affection, if the gaseous distention be first relieved by the use of the trocar. I think it better to give small repeated doses of one-half to one grain rather than one large dose.

More recently still our attention has been called by Prof. Dieckerhoff to the use of barium chloride as a purgative for the horse, and much is claimed for it in promptness, efficacy, and safety, although its advantage over eserine except in cost is not very clear.

It must constantly be borne in mind that purgatives are unnecessary in this affection, and that unless their use be very judicious they do more harm than good. I long since abandoned almost wholly the use of purgatives in colic from overfeeding, and can say that my success has been far better than under the usual regime. With the abandonment of purgatives, opium and morphine have, as already implied, also been discarded. If morphine is used, purgatives may be necessary to overcome its effects, but they are not essential in combatting the disease. I would then say if purgatives are to be given, use them only in the very early stages, in the absence of great gaseous distention, and select preferably eserine. If purgatives have not been used we frequently find, after the crisis of the disease has passed, or even during the early stages, a more or less severe diarrhœa which must not be checked but merely watched and controlled. Demulcent gruels, stimulants, and carminatives will almost always suffice, and if the feces are very fetid, salol should be added.

In colic due to the indigestion of musty or otherwise unhealthful food, leading to its early decomposition within the alimentary canal, we generally find a degree of tympany which necessitates early and repeated use of the trocar. Our efforts should next be directed toward staying the putrefaction of the intestinal contents by the administration of antiseptics, antiferments, diffusible stimulants, and carminatives. Salol and possibly naphthaline should prove highly useful during disease and convalescence. Opium is clearly contraindicated, as it serves only to stop the peristaltic action of the bowels and the secretion of digestive fluids, thus encouraging the decomposition of the food which constitutes the essential factor in the disease. Hepatic stimulants, increasing the flow of bile, stimulate healthy peristalsis and render the intestinal contents aseptic.

In the latter stages, frequently early in the disease, there is a marked tendency to diarrhœa which should be combatted by demulcent gruels, carminatives, and diffusible stimulants.

In colic due to a prolonged deprivation of food we frequently find coincident fatigue. Opium and morphine are rarely if ever indicated, but rather carminatives and diffusible stimulants, such as aromatic spirits of ammonia, ginger, alcohol, and nitrous ether, in the form of a drench with large volumes of warm water or gruel. The patient should be kept as quiet and comfortable as possible. Heat applied to the abdomen by means of hot wet blankets may afford much relief. Purgatives are clearly inadmissible.

In the colic of impaction in adult horses, due to the ingestion of food composed too largely of woody fibre, we are confronted with conditions requiring much time and not infrequently great tact to remove. There is plenty of time, so we need not hasten. Purgatives are almost universally prescribed, but the very drastic members of the class, such as croton oil, gamboge, eserine, and pilocarpine are clearly contraindicated, but we must rely rather upon those cathartics which will slowly and gently soften down and render pultaceous the accumulated dry mass, when little, if any, stimulation to the peristaltic action will accomplish its expulsion. The impaction generally occurs too far forward to render enemata of avail. Aloes serves the purpose fairly well, but large and repeated doses of the bland oils do better, and better still, perhaps, one moderate dose of aloes combined with oil, the latter to be repeated frequently.

The formation of gases rarely occurs. The pain is seldom severe, and calls for remedial treatment but rarely. When relief from pain becomes necessary, aconite, belladonna, or cannabis indica should be used, or if used with great caution morphine may be employed in doses of one-third to one-half grain subcutaneously, as in these doses it is not constipating.

In newly-born foals we frequently meet with obstinate colic, the result of retained meconium, or a little later owing to the ingestion of hay or straw. In these cases while large doses of bland oil may naturally aid, enemata of warm water or oil constitute the most reliable remedial measures. By fitting the nozzle of a bulb-syringe into an ordinary gum horse-catheter the latter can be slowly and carefully introduced up into the obstructed bowel, the water or oil being all the time slowly introduced. In this way the catheter becomes insinuated between the fecal mass and intestinal walls, following the flexures of the intestine the full length of the catheter and permits the fluids to mingle with and soften down the hard mass so that it readily passes away. Repeated enemas for several days or even a week may at times be necessary, the foal in the meantime being muzzled if needed to prevent the further ingestion of indigestible matters. In these the colic is rarely so acute as to warrant anodyne remedies.

In that form of colic due to obstruction of the rectum of young animals at pasture by a gummaceous feces, the cause of which we do not understand, the eversion of the rectum generally attracts more attention than the accompanying colic. It is to be remedied by the manual removal of the gummy feces, aided by enemas of warm water, and its recurrence prevented by a change in diet.

The colic of intestinal paralysis is closely allied to that of impaction in many cases, but calls for a rather more delicate judgment in its treatment. When the paralysis is due to a want of general tone in the animal, the result most frequently of inactivity and high feeding, we generally need only to wait, which is frequently about the most difficult of all courses to pursue, especially if the owner be present. Purgatives must necessarily increase the danger of the disease. Oleaginous laxatives and demulcents, by softening the intestinal contents, tend to relieve the malady. The pain is rarely extreme, and, when necessary, should be controlled by such remedies as will not interfere with normal intestinal functions. Small laxative doses of morphine frequently act nicely, relieving the pain and tending to overcome the disease. In one of my cases of a heavy draft-mare, in high condition and idle, one-half grain doses of morphia, repeated two or three times daily, controlled the colic perfectly, and on the sixth day, without other medication, the bowels moved normally, and recovery was complete without purgation, while purgation would probably have led to superpurgation with unfavorable results. In fact, my observations of the use of morphine in intestinal paralysis suggest strongly its use in severe impaction.

The treatment of volvulus and intussusception must be unsatisfactory, owing to the difficulty of exact diagnosis. In the present state of our knowledge it should be practicable, where positive diagnosis is possible, to give relief surgically. In cases where they are suspected and surgical correction not undertaken only palliative means are to be attempted, such as the relief of pain, prevention of gaseous distention, and abstention from purgatives.

There is much diversity of opinion as to the causes of these displacements, some ascribing them to the irregular muscular contractions of colic, others to the contractions induced by purgatives, and others in cases of volvulus to the rolling of the animal during the agony of colic. The important point to consider in our present theme is the possibility of producing or preventing them during our care of a colic patient. Volvulus is largely attributed by writers to violent rolling, but strangely enough apparently occurs most frequently, according to statistics furnished by these writers, where the rolling is prevented as far as practicable; so that others, notably Friedberger and Fröhner, in whose opinion I concur, think this has been given too great prominence. In fact, rolling is quite as likely—I think more likely—to correct such displacements than to cause them.

In a practice of sixteen years without recorded numbers, but perhaps light in colics, in which I have habitually made post-mortem examinations when practicable, I have found but one case of volvulus, that involving the entire cæco-colic mass, in which case from the history and symptoms the displacement must have occurred prior to symptoms of colic, and the rolling certainly had nothing to do with it.

Of intussusception I have observed three cases. One in an adult, affecting the small intestine, the history unknown, probably antedating the colic, and not due to purgatives; the other two were ileo-cæcal, in young foals, cases which I have already recorded, due to thrombo-embolic colic, the result of the presence of *strongylus armatus* in the mesenteric arteries. Friedberger and Fröhner allude to this as a common cause of intussusception. From all the evidence at hand it seems improbable that intussusception is caused by rolling, nor can we believe by regular contractions due to purgatives. It seems probable that it is due to irregular intestinal contractions, such as exist to a degree in colic, more perhaps in colic interfered with by injudicious doses of morphine combined with purgatives. I believe it quite rational to assume that such irregular contractions would ensue when colic is interfered with by conflicting treatment, by giving two remedies, like opium and aloes, the one in paralyzing doses, the other in doses to excite peristalsis. This precaution, with as nearly perfect freedom of the patient as possible, and care to prevent gaseous accumulations, we believe will do all that is possible to avoid the dangers from these accidents.

In herniæ and pressure from tumors we must rely upon surgical interference, but these operations may be made easier, safer, and in cases only possible by the previous use of narcotics or anæsthetics.

In colics due to thrombi or emboli we have an obstruction to the blood-supply, which must be relieved by the establishment of sufficient collateral circulation before recovery can take place so our treatment is purely expectant; and the result must depend upon the success or failure of the required collateral circulation before fatal lesions have occurred in the affected parts. We must rely upon those remedies which relieve the pain without interfering markedly with peristalsis, although in most cases it may be safely diminished for a time. Morphine in small or medium doses is indicated unless tympany is present, in which case it should give way to cannabis indica or to chloral, alcohol, and nitrous ether, the gaseous accumulations being studiously relieved by the use of the trocar. Purgatives are clearly inadmissable.

In spasmodic colic, while carminatives, stimulants, sedatives, and other classes of remedies relieve in many cases, morphine certainly comes most rationally into use as the chief remedial agent, and it may be pushed to a very considerable degree. It should constantly be borne in mind, however, that *morphine is not soporific in solipeds, but on the other hand in large doses is*

*uniformly and dangerously excitant*, as has been pointed out by Guinard (*Journ. de Med. Vet. et de Zoötechnie*, July, 1893), so that it must be used cautiously and not pushed beyond the degree needed for the alleviation of pain. No remedy is so misused, and its actions so misunderstood by veterinarians as is opium and its chief alkaloid, morphine. We are generally taught that it is soporific and depressant in a horse. So when the veterinarian makes too great haste to relieve pain by its use the horse becomes excited, nostrils dilated, eyes staring, tail elevated, and the patient becomes uncontrollable, the victim of opium-poisoning, while the improperly taught veterinarian tries to overcome this excitation by larger doses, adding more and more to the difficulty, finally ending in fatal opium-poisoning, and the veterinarian is left wondering why his morphine would not quiet the patient. Not long since I read the communication of a layman of how the veterinarian in his locality habitually lost all colic cases because morphine failed to quiet them, but instead they were more restless and moved faster as more of the drug was given, while other horses treated by novices with sage-tea or peppermint recovered, and drew therefrom the conclusion that, so far as colic is concerned, veterinary science is a conspicuous failure, and there was much logic in his conclusion. I recently saw a horse very much excited by one grain of morphine given to relieve pain after castration, and to relieve this excitement two more were given, which rendered the patient utterly uncontrollable and dangerous to approach, and it was only with the greatest difficulty that chloroform was administered and a check placed upon a very serious-looking case of opium-poisoning. So while we commend morphine in spasmodic colic we caution judgment. Owing to the generally empty condition of the digestive canal purgatives are uncalled for, and as already stated should on no account be given if opium has been employed to relieve the pain. Cannabis indica is preferred to morphine by many in these cases, and it certainly offers some points of superiority. Hot fomentations and stimulating applications to the abdomen are probably of more service in these than in other colics.

Colic due to external mechanical pressure, such as being accidentally or intentionally cast in a cramped attitude calls for little remark as to treatment beyond the very obvious suggestion to place the animal in a comfortable position. The fact that colics do arise from casting should suggest care in our methods to avoid cramping the limbs and body as far as possible, especially for operations of long duration. We should be careful, too, that colic patients do not have their malady aggravated by being left cast in an uncomfortable position, and also we should bear in mind that probably a not insignificant number of colics are due primarily to ill-fitting, uncomfortable harness and improper hitching, by which the body is thrown in a strained position for a long period.

In cases where colic may be traced to presence of parasites in the digestive canal we generally find coincident debility, thus suggesting stimulants, carminatives, and bitter tonics, combined with vermifuges, which latter should be followed by an aperient, preferably of oil, while in some cases where the patient's strength will permit it a brisk purgative may be substituted with advantage.

The presence of calculi in the digestive canal are rarely diagnosed during life. They are then to be treated, if not by surgical interference, by oleaginous aperients, and if available by warm enemata, in order to soften the fecal matter and relax the intestine in which the calculus is imprisoned. Drastic purgatives should not be employed as a rule, as their violent action may result disastrously, but if to be used at all it should be done early, before the affected parts become paralyzed and inflamed.

Colic due to the ingestion of sand should be treated with a view to expelling the sand gently and allaying irritation of the bowels by means of moderate quantities of bland oil and demulcent gruels.

In general, we would say in all forms of colic see first that no remedy administered can in any probable manner exert an unfavorable influence upon any pathological condition present. Until we have clear evidence of their necessity, we should always avoid purgatives and agents which induce intestinal paralysis, and in all events avoid the combination of the two if either is desired. It should be remembered constantly that purgatives are rarely directly necessary or advisable ; but commonly necessitated or supposed to be by the improper use of opium, a drug which annually kills ten horses with abdominal diseases for each one it saves.

